# ‘Keeping your body and mind active’: an ethnographic study of aspirations for healthy ageing

**DOI:** 10.1136/bmjopen-2015-009973

**Published:** 2016-01-07

**Authors:** Cornelia Guell, Guy Shefer, Simon Griffin, David Ogilvie

**Affiliations:** 1 MRC Epidemiology Unit and UKCRC Centre for Diet and Activity Research (CEDAR), University of Cambridge School of Clinical Medicine, Cambridge, UK; 2 The Primary Care Unit, Department of Public Health and Primary Care, University of Cambridge, Institute of Public Health, Cambridge, UK

**Keywords:** QUALITATIVE RESEARCH, Physical activity, Ageing, Social context

## Abstract

**Objective:**

To describe and explore perceptions, practices and motivations for active living in later life.

**Design:**

Qualitative study with semistructured interviews and ‘semistructured’ participant observations of participant-selected activities, such as exercise classes, private or organised walks, shopping and gardening.

**Participants:**

27 participants (65–80 years) from the European Prospective Investigation into Cancer Norfolk study, purposefully selected by gender, age, occupational class, living status and residential location; 19 of the participants agreed to be accompanied for observed activities.

**Setting:**

Participants’ homes, neighbourhoods, places of leisure activities and workplaces in Norfolk, England.

**Results:**

All participants regarded a positive attitude as important for healthy ageing; this included staying active, both physically and mentally through sedentary activities such as reading and crosswords. ‘Getting out of the house’, being busy, or following a variety of interests were regarded as both important motivators and descriptions of their ‘activeness’. Purposeful activities formed an important part of this, for example, still being engaged in paid or voluntary work, having caring responsibilities, or smaller incidental activities such as helping neighbours or walking for transport. Many also reported adapting previous, often lifelong, activity preferences and habits to their ageing body, or replacing them altogether with lower impact activities such as walking. This included adapting to the physical limitations of partners and friends which dictated the intensity and frequency of shared activities. The social context of activities could thus form a barrier to active living, but could also encourage it through companionship, social responsibilities and social pressures.

**Conclusions:**

Promoting and maintaining physical activity among older people may require more attention to activeness as an attitude and way of life as well as to its social context, and initiatives encouraging broader activity habits rather than discrete activities.

**Strengths and limitations of this study**

- This qualitative study used the innovative method of ‘semistructured’ participant observation together with interviews to explore aspirations and daily practices of active living among older people.
- Rather than being experienced as discrete behaviours in everyday life, ‘activeness’ can form part of more general aspirations of ageing proactively.
- The study provides a useful insight into how social support can be garnered as a facilitator of active living, gaining encouragement, companionship or purpose.
- The social context can also be a barrier that needs to be negotiated, for example, when the ill-health or physical limitations of others reduce the time available for activeness or the intensity of activities that can be performed together.
- The main limitation of this study was that the sample comprised participants whose views might have been shaped by their long-time participation in a health-related study; their narratives can, however, serve as case studies of proactive strategies that others could adopt.

## Introduction

A recent appraisal of the new WHO physical activity guidelines^1^ included a perspective on older adults, who are known to find it difficult to meet targets for moderate and vigorous-intensity activity, and proposed a more achievable message of reducing sedentary time and integrating more light activity into everyday life.^2^ Levels of physical activity decrease in later life, at a time when primary, secondary and tertiary prevention of chronic conditions such as diabetes, cardiovascular disease and cancer is increasingly important.^3–5^ Physical activity has significant health benefits for older adults, improving physical and mental health, mobility and independence.^6–8^ However, interventions to promote physical activity for older adults tend to be exercise-based programmes framed within individualistic psychological behaviour change models, and have typically produced only small or short-lived changes.^9^ ^10^ A broader focus on active living^11^—including leisure, household, transport and occupational physical activities—and on social influences^12^ might offer a more promising and effective public health strategy to promote physical activity by integrating it into everyday life.

The Five Year Forward View for the UK's National Health Service calls for a ‘radical upgrade of prevention’ as well as supporting self-care by recognising patients’, families’ and carers’ motivations in life and valuing them as ‘experts by experience’.^13^ ^14^ Qualitative research can provide valuable insights to help understand how best to develop, promote and support such preventive strategies by exploring people's aspirations to be active and their social contexts.^15^ Much of the literature has focused on structured exercise, with only limited consideration of other (what are sometimes referred to as ‘unsupervised’) physical activities, mainly walking,^16^ and little is known about perceptions, practices and motivations of active living more generally at any life stage. Those who have investigated physical activity beyond participation in exercise have shown an increasing interest in the physical and social environments in which these activities take place.^17–19^ However, most qualitative research in this area has used a limited set of methods, predominantly interviews and focus groups.^16^ It has been suggested that studies could benefit from integrating more spatial methods to allow for fuller and more nuanced descriptions of environmental factors,^20^ with photovoice proving a particularly popular method.^21^ ^22^ Similarly, it could be argued that the use of qualitative observational methods enables a more in-depth appreciation of the complex social context, as well as other aspects of the environment that participants may not reflect on explicitly in interviews. In this study, therefore, we aimed to investigate and describe in depth how active living relates to later life experiences and to aspirations and strategies of healthy ageing, using an ethnographic research design that combined interviews with participant observation.

## Methods

### Research design

The ethnographic research design was framed within social theory, understanding active living as social practice, which assumes that individual behaviour shapes and is shaped by social context.^23^ We therefore combined semistructured interviews about life history, articulations and motivations of active living, with what we call ‘semistructured’ (time-bound, one-off) participant observation. The observations aimed to explore social interactions, unarticulated barriers to active living, and the environmental context of activities.^24^ All participants gave their informed written consent.

### Setting and participants

The study was set in Norfolk, a largely rural county in the east of England, including the city of Norwich. Purposeful sampling aimed for a diverse, information-rich sample of participants with experiences that might vary by their gender, age group (65–69, 70–74, 75–80 years), occupational class (professional or manual), living status (alone or cohabiting), residential area (urban or rural, in neighbourhood clusters), and physical activity level (table 1).^5^ All participants had to be physically independent. We recruited participants from the European Prospective Investigation into Cancer (EPIC) Norfolk study, a cohort of initially 25 639 men and women selected between 1993 and 1997 from the general population then aged 45–79 years.^25^ Recruited through general practitioner surgeries, EPIC-Norfolk participants completed questionnaires on their diet, lifestyle and health after 18 months, 3 years, 10 years and 13 years, and also attended four health checks. For this study, we recruited participants from among those who had attended the fourth health check (4HC, which included about 3000 participants at the time of recruitment). This health check included the use of accelerometers to measure physical activity over a period of a week. These accelerometer counts were used to group 4HC participants into deciles of activity level, from which we sampled participants belonging to the more (but not the most) active levels, or the more (but not the most) inactive levels (table 1). Initially 32 potential participants were contacted by mail by the EPIC-Norfolk study coordination team, and 22 agreed to take part; another 8 were invited in a second round of recruitment to fill under-represented categories (inactive, manual occupational class and oldest age group), of whom five responded positively (68% response rate). Nineteen of the 27 participants agreed to take part in a follow-up participant observation activity (70% response rate). In the final sample of participants, we therefore achieved a relatively even spread across our sampling criteria (table1). During the interviews, 19 participants also volunteered information about their health. Seven reported various musculoskeletal conditions such as osteoarthritis that limited their mobility; seven reported living with other chronic diseases (cancer, diabetes or cardiovascular disease); and six reported limiting conditions of their activity partners such as their spouses or children (of whom one also reported a limiting condition of their own).

**Table 1:** Participant characteristics

	N
Gender*	
Male	15
Female	12
Age (years)*	
65–69	10
70–74	7
75–80	10
Activity level*^,^†	
Active	14
Inactive	13
Occupation‡	
Professional (I, II, IIIN)	14
Manual (IIIM, IV, V)	13
Living status*	
Cohabiting (married or living with partner)	13
Living alone (single, widowed, separated, divorced)	14
Residential area*	
Rural	15
Urban (or urban sparse)	12
Total	27

*From European Prospective Investigation into Cancer (EPIC)-Norfolk fourth health check data (2013–2015).

†From Actigraph accelerometer counts, decile 7, 8, 9 for more active group, decile 2, 3, 4 for more inactive.

‡From EPIC-Norfolk baseline data (1993–1997).

### Data collection

Two experienced ethnographers—a female anthropologist (CG) and a male sociologist (GS)—collected the data between September 2014 and March 2015. Twenty-seven semistructured interviews, lasting 20–60 min, were conducted in participants’ homes, flexibly using a topic guide with questions about their everyday activities and motivations; their lifestyle opportunities, choices and motivations across the seasons and their life course; and their aspirations into older age (box 1). All interviews were audio-recorded, with accompanying notes taken, and transcribed verbatim. The researchers and participants had not met before the interviews, but initial rapport established during the interviewing was important for the subsequent participant observation sessions which were undertaken with the same researcher.

Box 1 **Interview topic guide**

**A: Descriptions of active and sedentary living—*experiences***

- *Types of activities*: Can you describe your regular activities for me?

*Prompts*: ‘Typical day in your life’; activities can be more or less active; leisure, work/volunteering, travel; longstanding, new; frequent (eg, daily newspaper reading) and infrequent (eg, holiday cycling); typical journeys.

- Rating activities
  Can you tell me a bit more about these activities?

*Prompts*: Which ones are important activities (eg, weekly shop, grandchild care, volunteering), favourite activities (eg, gardening), preferred activities (eg, walking, TV watching), required activities (eg, housework, dog walking)?—And can you tell me why this is so?

- Do you think you are a relatively active or inactive person?

*Prompts*: Why? Examples: comparison with other people; looking back through life course.

- *Activities over the life course*: Thinking back through your life, what were typical past activities in your past?

*Prompts*: Starting with childhood, as a young adult, during transition to work life/family life, and transition to retirement.

**B: Social context of active and sedentary living—*opportunities, choices***

- *Activities with whom*: Can you tell me with whom you do these activities?

*Prompts*: For each activity of A: family, friends, strangers, alone.

- *Activities for whom*: Are you doing any activities for other people? Can you give me some examples for whom and which activities?

*Prompts*: Which regular activities, for whom; for example, caring for grandchildren or spouses; help the community; to support activities of others; or for yourself: ‘me-time’?

- *Activities because of whom*: Can you tell me a bit about who was or is involved in deciding on activities?

*Prompts*: Upbringing; invited by others; prescribed; pressured, encouraged, supported.

- *Activities where*: Where are you doing these activities? Can you tell us a bit about these places?

*Prompts*: Home, community; free/cost money; outdoors/indoors; good (pretty, healthy, green, comfortable) and bad (ugly, unsafe, grey, inadequate) environments/places/spaces.

**C: Attitudes towards active and sedentary living relating to health and well-being—*motivations***

- *Ageing*: What are expectations in our society of ‘ageing well’? What are your personal goals/expectations/plans?

*Prompts*: How do these relate to each other; how does this relate to health, well-being, quality of life; barriers and facilitators to ageing well?

- *Benefits of physical activity*: What role does an active lifestyle play for you?

*Prompts*: For you personally, for older people in general, for all age groups in general; physical (independence, mobility, primary/secondary prevention of chronic conditions), mental (well-being, also independence), social (also independence, camaraderie, companionship).

- *Benefits of sedentary living*: What role does an inactive lifestyle play for you?

*Prompts*: For you personally, for older people in general, for all age groups in general; rest, relaxation; avoiding injury, exhaustion.

Nineteen participants agreed to be visited again to be accompanied in active or sedentary activities of their choice. Participants and researchers discussed the purpose of these observations at the end of the interview in the course of selecting the activity and organising the visit. We explained that we did not aim to assess the quantity or intensity of physical activity involved in the activities, but rather to find out about the social and physical environment in which these were taking place. Although few participants chose a primarily sedentary activity for observation, we were, nonetheless, able to observe sedentary activities as these were often linked to more active pursuits, such as having a cup of tea or coffee before or after a walk or exercise session. Observations lasting 1–3 h included structured exercise such as yoga, active home console games, walks for leisure and transport, dog-walking, organised group walks, shopping trips, gardening and more sedentary activities, such as art class, luncheon club, concerts, meditation, drives and bus rides, coffee and tea breaks and visits to work places. A total of about 30 h of observations and informal conversations were written up in ethnographic field notes.^26^

### Analysis

Data collection and analysis were conducted iteratively and simultaneously. The thematic data analysis involved familiarisation with the textual data through repeated reading, and identifying codes and larger thematic categories, which were then synthesised and written up as themes. The coding was guided pragmatically by the research objectives but also allowed for inductive analysis of unanticipated topics or meanings. Particular attention was placed on constant comparison between cases and on negative cases to ensure rigour in the analysis. Data analysis was aided by use of the qualitative data analysis software NVivo V.10.^27^ The ethnographic field notes served to triangulate the findings; they were not openly coded like the transcripts, but used to help at the later stage of analysis when codes were synthesised into categories, further exploring how categories played out differently in different situations or participants. Further interrogation of the qualitative analysis was possible using the EPIC-Norfolk quantitative data; in particular, we compared participants’ descriptions and perceptions of activity levels with their objectively measured data. To strengthen the analysis and ensure trustworthiness of the process, five sample transcripts were double coded by both researchers; the emerging code book was discussed iteratively throughout the double-coding exercise; and emerging categories were discussed extensively between the two researchers and the larger study team throughout the data collection and analysis. Finally, preliminary results of the analysis were presented to the participant panel of EPIC-Norfolk, at which non-identified participants of this qualitative study were also present, and interpretations were discussed and confirmed in open debate.

## Results

We present a summary of findings on participants’ perceptions and motivations of active living (aspirations), their descriptions of how they integrated ‘activeness’ into their lives (practices), and the social context in which active living was shaped. Original illustrative quotations are summarised in box 2.

Box 2 **Participant quotes**

**Aspirations of active living**

Positive attitude

- ‘It is an attitude, yes, you know, provided you can, you're healthy enough … but while I'm healthy and fit then I'll do as much as I can. … I mean we were talking the other day about it and somebody was saying since a couple they knew retired all they seem to do is sit in front of the television waiting for the undertaker, you know, which is awful *[laughs]* …. So no, I do try and keep active and keep mentally active as well.’ [Participant 14, woman, 75–80, active, professional, rural, living alone]
- ‘I think it's very important to find something you love, you have to have a passion, you have to have a bit of movement and keep your body moving.’ [Participant 5, woman, 65–69, inactive, professional, urban, living alone]

Keeping body and mind active

- ‘I think you've got to make an effort to keep fit and to keep you know, I think if I don't keep active I might seize up altogether, and I try and get my brain active, I like doing Sudoku puzzles … because I think that's good.” [Participant 16, woman, 70–74, active, manual, rural, cohabiting]
- ‘Keep active, that doesn't mean to say you've got to play tennis or golf … but keep the body moving even if it's doing a bit of gardening for half an hour every day and make it every day, walk for your paper, don't get in the car …, but most of all keep the brain active, yeah, keep the brain active, even if it's just meeting up with a group of friends and have a discussion…’ [Participant 20, man, 75–80, inactive, manual, rural, living alone]
- ‘I think you've got to do things. It's the easiest thing in the world just sitting there, just read or do something. You've got to get out; you've got to move about. And yesterday was the first time I've really done anything since I've had the heart attack, and, oh, it was lovely, you know, just to, probably clear a few leaves and burn stuff, and had the dog running around again, like he used to, you know, it was just nice.’ [Participant 23, man, 75–80, inactive, manual, urban, cohabiting]

Tensions between staying busy, and active or staying fit

- ‘I can still run down a road with no real problems. I'm a little bit overweight, and I should get … some of that weight off because … I'd probably be able to walk around a lot better than I do, but I don't have the time *[laughs]*, you know, it's do you keep fit or do you keep active, I don't know. As I say I know what I should do but *[laughs]*, not easy.’ [Participant 17, man, 70–74, inactive, professional, rural, cohabiting]

**Practices of active living**

- Being out and about
- ‘If I was home all day …, you know, I'd go loopy, I really would.’ [Participant 14, woman, 75–80, active, professional, rural, living alone]
- ‘I mean, my wife's a classic example there, I mean, she's getting all these sorts of things, arthritis and things, but she doesn't slow down.’ [Participant 23, man, 75–80, inactive, manual, urban, cohabiting]
- ‘I don't like sitting … and just watching telly or, I've got to be on the go. So … I do like every day, at least, to get out in the fresh air, have a little walk, even if it's only for an hour, just down the city, you know, or anything.’ [Participant 6, woman, 65–69, active, manual, urban, living alone]
- ‘… people talk about retirement but I'm as busy now as I was when I was working in many ways….’ [Participant 12, woman, 70–74, active, professional, urban, cohabiting]

Purposeful activities

- ‘… our only routine really is every Friday we look after our two grandchildren and we … look after them for the day, take them to school, pick them up and keep them safe ‘til their parents come home.’ [Participant 1, woman, 65–69, inactive, professional, rural, cohabiting]
- ‘I think I'm very lucky that I'm in a job that I do enjoy and can keep going as long as I want, you know. So yes, I think … it's [all about] being active doing the things you like.’ [Participant 14, woman, 75–80, active, professional, rural, living alone]
- ‘I do like to walk for a purpose if that makes sense, you know? Like to walk to the shop to get a newspaper.’ [Participant 22, woman, 65–69, active, professional, rural, cohabiting]

Adaptive activeness

- ‘I would have loved to still play tennis but I can't, I can't run. … I thought ‘I'm gonna save up and get that [Wii console]’, ‘cos I just enjoyed it, ‘cos in my head, you see, I'm really playing tennis.’ [Participant 15, woman, 75–80, active, manual, rural, living alone]
- ‘The walks have got less arduous obviously, rather than looking for high walks, you're now looking for lower walks…’ [Participant 11, man, 70–74, active, professional, urban, cohabiting]
- ‘…as the body shuts down, I don't mean that too literally, you know, so you don't play the tennis but you can still play bowls and you still can do your walking and I can still play with the grandchildren so physically I can do all those things still…’ [Participant 20, man, 75–80, inactive, manual, rural, living alone]

**Social context of active living**

Social motivators

- ‘Every fortnight, I have a friend I used to work with, and we go for quite a long walk. Maybe anything from sort of seven to fourteen miles we do on that.’ [Participant 7, man, 65–69, active, manual, urban, cohabiting]
- ‘…a dog is so much company especially if you're on your own, you know, it's lovely so yes, I do enjoy the dogs and it's something I'd hate to think I hadn't got one to walk, gets you up in the morning, gets you out, you've got something to think about.’ [Participant 14, woman, 75–80, active, professional, rural, living alone]
- Questions: ‘Do you think society expects of you to stay very healthy and very active?’
- Answer: ‘No, my wife expects me to stay very healthy…’ [Participant 11, man, 70–74, active, professional, urban, cohabiting]
- ‘… sometimes [taking care of the grandchildren] it's a bit of a chore and it's hard work after school because they're usually a little bit tetchy…, and I have to drive twenty-five miles to get there so it takes all afternoon when I could be doing something else.’ [Participant 5, woman, 65–69, inactive, professional, urban, living alone]

Social limitations

- ‘I think … this is as much due to probably to my wife as to me. We do … just about everything together … so part of my fitness is probably down to her, or lack of it, and vice versa, do you know what I mean?’ [Participant 17, man, 70–74, inactive, professional, rural, cohabiting]
- ‘For the last six, nine months my wife was not very well, I mean, so going out was an effort so we probably tended to go out less…’ [Participant 2, man, 75–80, inactive, professional, rural, living alone]
- ‘I do … any heavy work to do in the garden for example I'll do that. But she [my wife] likes to do her bit,…she's got quite bad arthritis and her hands are bad, … she's got a sort of sciatica problems with one of her legs and it forces her to walk very slowly and when I'm walking with her I find that difficult, it's because my natural gait is a lot faster and I find it hard to keep down to her, … yeah *[laughs]*, so that's a problem but it's one you have to face, you know.’ [Participant 18, man, 70–74, inactive, professional, urban, cohabiting]

### Aspirations of active living

#### Positive attitude

Most participants felt strongly about the need to have a positive attitude towards ageing. Adversities such as ill-health and increasing physical limitations were faced with a ‘getting on with it’ approach; good health was seen to be cherished and something to be made use of. Retirement or older age should not be an excuse for sedentary living, but, on the contrary, could be a chance to concentrate on activities that were enjoyed.

#### Keeping body and mind active

Most participants also said that the key to active ageing was to keep the body moving, as well as, crucially, to keep the mind active. Some participants suggested that these could be achieved in combination, for example, by engaging in activities that were both physically active and intellectually stimulating. Others highlighted that while physical activity was indeed important, sedentary pursuits—in particular, puzzles such as Sudoku or crosswords, but also reading, arts and crafts, or music—were equally vital to maintaining mental and cognitive health in later life.

#### Tensions between staying busy, and staying active or fit

Some participants’ descriptions of keeping active did not match their accelerometer counts. Others explicitly stated that their activities did not necessarily translate into physical activity or fitness. In fact, many participants talked about leading a busy and engaged life in retirement that rivalled the time constraints in their previous working life, and could be a barrier to being physically active.

### Practices of active living

#### Being out and about

Participants described being active in complex ways; this could mean ‘not sitting down’ or ‘getting out of the house’, leading a busy life or having a variety of interests or responsibilities, or being sporty or enjoying particular activities or clubs. Their practices of active living thus described what we came to refer to as ‘activeness’, a term chosen to reflect both actual activity and a disposition to being active. This active way of living was rarely described in terms of discrete activities, but rather as many ‘parcels’ of activity, some active, some sedentary. The participant observations, in particular, highlighted that active and sedentary activities were often intertwined, for example, leisure walks might include a pub lunch.

#### Purposeful activities

Many activities incorporated incidental physical activity, but this did not seem to be the main reason for engaging in these activities; instead, they were more likely to be undertaken for such purposes as socialising, working, caring or transport. In fact, caring was described as an important source of activeness. Also, half the participants were still working, either continuing paid full-time or part-time work or volunteering. Somewhat surprisingly, 5 of the 19 participant observations included visits to workplaces. People also described the need to be active ‘for a purpose’ in smaller ways, such as walking to buy a newspaper rather than merely walking for leisure.

#### Adaptive activeness

Many participants experienced ill-health or increasing physical limitations and described various ways of adapting to these. In particular, those who reported lifelong activity habits seemed to show great motivation to adapt them. Some chose lower impact activities, for example, by moving from tennis to short tennis or even to game console tennis, or from cricket to bowls (box 3 for case study). Others replaced previous activities altogether, mainly with walking.

Box 3 **Participant case study for adaptive activeness**

Keeping the ball up: Susan's story

The following case shows the complex ways in which ‘adaptive activeness’ is tackled. Susan is 77 years old, and has been living alone in her rural detached home since her husband passed away. In our study sample, she belongs to the more active group. She has been sporty all her life, growing up in a family that avidly played tennis. She had to stop playing at her club when her husband required care and more of her time. Her other lifelong passion had been her dogs and walking. When she was widowed, her dog continued to be a reliable and comforting companion on her walks. By the time of the interview and observations, however, she had also lost her dog. She now considers herself very inactive; her children and grandchildren do not live close by, and she feels trapped in the house. But she has found an activity that she can do indoors that enables her to revisit her former pastime. Her grandchildren have introduced her to a gaming console on which one can play tennis. The game enables her to keep up her skills in coordination and balance while enjoying the challenge and sensory pleasure (‘the sound of the ball bouncing and hitting the racquet’). Susan invited us to join her for a morning of playing these games. The games available include her favourite tennis but also golf and other sports, and she recently got a set of balance games, which she appreciates for improving not only her—increasingly deteriorating—sense of balance, but also her leg strength and concentration. While these console games are clearly an indoor activity and did not help Susan to get out of the house, they are fun games to play with companions, and Susan has a friend who joins her regularly.

### Social context of active living

#### Social motivators as facilitators

Having access to, or providing, social support seemed to encourage active living; this was an experience narrated not only by those participants who were cohabiting, but also by those living alone. Participants talked about family, friends or neighbours as sources of encouragement or company in activities, or motivators to stay healthy. Dogs were also appreciated as both a reason for walking and social company when walking. Social motivators for active living related not only to friends and family providing support, but also less tangibly to social norms and pressures. Some participants spoke of housework and gardening as chores that were kept up because of social expectations of tidy gardens or households. Caring for grandchildren was also seen as a source of physical activity, undertaken not necessarily for pleasure, but sometimes as a chore that was expected of them.

#### Social limitations as barriers

While social obligations and ties were a source of physical and social activeness, they could also limit active living. The physical limitations and ill-health of others (spouses or activity companions) acted as barriers to being active, or to being active at a frequency or intensity desired by some participants and suitable to their level of fitness or health. Participant observations, for example, included spouses and other family members, and in three observations the low activity level was shaped by these companions’ difficulties with walking (box 4 for case study). Finally, social norms and pressures also seemed to act as barriers to active living, most notably when participants felt their limitations would restrict others. For example, a person might leave a walking group for fear of holding others back. Social responsibilities—and an expectation of others that retirement meant flexibility and availability—could also leave little time for other activities and interests.

Box 4 **Participant case study for social limitations**

Finding one's stride: Peter's story

The following case shows complex ways in which active living in later life is shaped by social context. Peter is 68 years old and lives with his wife in their suburban detached home. He also belongs to the more active group in the study sample. Peter has led an active life, starting during his career in the army and continuing through many subsequent, often manual, jobs until his early retirement. Peter spends his retirement in many different activities, for example, attending to his garden and helping a friend at his allotment, and socialising with friends and family. His grandchildren require looking after once a week, and they also visit his father-in-law regularly to provide support. His favourite pastimes and lifelong habits are walking and bird watching. For the study's participant observation, we were invited for a walk to and in a nearby country park. The observation started with a walk through the city to the outskirts—Peter often walks for transport because he cannot drive—where we were joined by his wife who drove to join us at the park. She struggles to walk because of a persistent foot problem, and we had to stop walking briskly to accommodate her. The park was chosen for this outing for its even paths and easy opportunities to rest. For longer walks on uneven terrain Peter meets with a group of friends. These walks, however, have also become increasingly slower and shorter, and the subsequent pub lunch longer, because members of the group increasingly experience joint problems and other ill-health. Despite these challenges, Peter seems to have found strategies to keep up his walks; physical activity, however, seems to be an incidental side effect of his favourite pastime.

## Discussion

### Principal findings

The participants in this study shared strong normative perceptions that healthy ageing should be actively pursued. Notably, this included not only physical activity but also keeping the mind active to maintain cognitive health. This kind of ‘proactive’ ageing seemed to be framed by strong norms of sedentary living as socially undesirable. It also seemed to represent a strategy of deliberately and determinedly addressing fears of old age such as frailty, dementia or social isolation.

Active living was mostly described as a non-discrete, unstructured way of living, a cumulation of a variety of activities that were more active for some, more sedentary for others. We conceptualised this as ‘activeness’ to capture the complex interplay of disposition, lifestyle and actual activities.^28^ Levels of activeness also depended on experiences of ill-health and physical limitations of one's own ageing body or those of others, and participants narrated various strategies by which to overcome these to maintain their habits and interests.

Central to this active pursuit of ageing was the social context. Socialising with partners, family and friends was both a motivator and a goal of active living. Social responsibilities and norms instigated activeness through chores, caring for or helping others. Social ties and norms, however, could also limit levels of activeness, for example, by constraining time for activities, or discouraging participation in group activities that were deemed too challenging.

### Results in context

A recent systematic review of qualitative studies of physical activity in older age also highlighted social influences in its thematic synthesis of findings, identifying social interactions and encouragement as important facilitators for physical activity in this age group, whereas social awkwardness and social responsibilities could act as barriers to physical activity participation.^16^ A majority of the studies included in that review were narrowly focused on structured exercises. Only the more recent studies had adopted a broader focus, examining other types of physical activity and a wider set of mechanisms related to social factors, for example—as we found in our study—that responsibilities such as caring for others could also be a source of incidental physical activity rather than a barrier.^29^ However, it remains the case that little is known about perceptions and practices of non-structured physical activity, and few studies have capitalised on the range of qualitative methods to explore deliberate and incidental active living. Studies taking a broader focus on active living have generally echoed our findings that continuing lifelong activities (albeit adapted when necessary) was an important motivator, as was the notion that activities should be purposeful.^30–32^ Furthermore, it seems important that activities can be integrated into everyday life.^33^ Using ethnographic methods, we were able to observe and discuss in depth with our participants their ordinary everyday activities as contributors to active living, and the interplay of aspirations of healthy ageing, lifelong ways of living and social context. While many studies have found healthy ageing to be a motivator for staying active, and physical limitations and ill-health to be a barrier to realising these ambitions,^16^ to the best of our knowledge, our study is the first to observe and describe participants’ challenges in adapting not only to their own physical limitations in later life,^16^ but also to those of their activity companions.

In describing the complexity of social facilitators and barriers, we also contribute to a nascent body of social science research that understands physical activity not as a discrete health behaviour but as a set of practices in their social context. Using theoretical frameworks of ‘social practice’, social scientists argue that promoting behaviour change requires understanding the social worlds, lifelong habits and aspirations, *and* environments in which behaviours take place.^34^ ^35^ In other words, it is not merely identifying a set of barriers and facilitators, but understanding the way these are linked—as social practice—that may help to bring about change. For example, a qualitative network analysis mapped motivations of active living in everyday life in Belfast as a ‘complex web of concerns, processes and events’—such as traffic, safety, the weather or actions of neighbours—that people negotiated when deciding to be ‘out and about’.^19^ In a previous study of active commuting, we described peoples’ decisions to cycle or walk to work as reflecting a tactical negotiation of their aspirations and experiences (eg, as lifelong cyclists, victims of traffic accidents, or seekers of ‘a bit of me-time’), and their social worlds (school runs, relocation, shift work).^15^ ^36^ Similarly, householders in four English towns made decisions about walking or cycling for transport based on their weighing-up of perceived risk, family responsibilities and reputation (what were judged to be acceptable modes of transport).^37^ In our study with older adults, active living included both active and sedentary activities, often in combination or related to motivations of healthy ageing, and firmly situated in and enabled and constrained by people's social lives.

While this emerging body of research, to which our study contributes, is largely qualitative and provides contextual and context-specific insight into physical activity, it complements current epidemiological investigations that increasingly focus on ‘bouts of activity’^38^ and on reducing sedentary behaviour^39^ and inactive lifestyles. These have provided evidence that small changes, for example, incorporating 20 min of walking into the daily routine, can have substantial health benefits, particularly in people classified as inactive or moderately inactive.^40^

### Implications for clinical practice and future research

Qualitative studies provide in-depth insight into people's aspirations, underlying social norms, and strategies to put these aspirations into practice. If disease prevention strategies aim to empower people to make healthy living choices, these studies can help tap into their experiences to develop appropriate and relatable messages—acknowledging them as ‘experts by experience’.^13^ An evidence review commissioned by the National Institute for Health and Care Excellence suggested that brief advice in primary care has a modest effect on increasing physical activity levels.^41^ Based mainly on self-reported measures of physical activity, however, the evidence for the effectiveness of this advice is at best weak and may be subject to social desirability bias.^42^ We suggest that reasons for the limited effectiveness of such brief message-based interventions may lie in a lack of attention to the circumstantial and social complexities that older adults face, which we describe in this study. Clinicians’ recommendations and public health interventions have to fit into people's everyday circumstances, constraints and challenges, and some people might already be trying to pursue more active lifestyles, but need some help to tackle the particular challenges they encounter. For example, one qualitative study found that while participants did not find advice from healthcare professionals to be useful in increasing their levels of physical activity, they did appreciate guidance on how to tackle particular mobility and health challenges in being active.^43^

We found that older adults’ active living practices transcended singular activities towards a more general strategy directed at achieving physically and cognitively active ageing by following a variety of interests and aspirations such as ‘getting out of the house’. As older adults can find it difficult to meet a physical activity target of 150 min a week of moderate intensity physical activity, and in view of a call to change the emphasis of such messages to reducing sedentary living,^2^ there may be potential for interventions aiming to substitute active activities for sedentary activities in small ways, for example, by incorporating more active travel into daily tasks, such as walking to shops for small purchases.^40^ To date, public health strategies to promote active living have tended to focus on changing the physical environment to support active travel; or on individualised approaches that allow for different ways of fitting activeness into everyday lives, such as pedometer-based interventions that allow for flexibility in how steps can be accrued within and outside the house.^42^ ^44^ A new focus on ‘activeness’ as an everyday way of living should encourage further development of intervention strategies to encourage ‘getting out of the house’ by providing opportunities for meaningful or social activities. Key in these interventions will be to give advice that is acceptable and feasible for older adults, for example, how to accrue incidental physical activity through such purposeful activities, or how to negotiate physical limitations within a social group. One challenge will be to encourage ‘active enough’ lives; as some of our participants noted, while ‘being busy’ can be a great motivator for being active, a busy life can also easily preclude being sufficiently physically active. Another challenge will be to integrate messages about the value of reducing sedentary lifestyles with related but slightly different evidence that recommends particular levels of moderate or vigorous physical activity as necessary for effective healthy ageing.^45^ ^46^

Finally, asking patients about their social lives and circumstances should not only be part and parcel of clinical encounters, but also integral to public health research. Ethnographic studies like this, combining interviews with observations to reflect on and observe social context, should be complemented and corroborated by larger scale quantitative investigations using social network analysis and related approaches. These can, for example, measure the effects of structural and functional components of social relationships on health practices, and inform individual and population-based prevention strategies.^47^

### Strengths and weaknesses of the study

This qualitative study aimed to research active living ‘actively’, combining in-depth interviewing with observational data of participant-chosen activities to arrive at more in-depth, ‘thick descriptions’ and understandings of participants’ experiences.^24^ This research design uncovered previously unexplored challenges in physical activity in older age, such as limitations of activity companions. That said, the pragmatic choice of ‘semistructured’ one-off and time-limited observations broke with the anthropological tradition of ‘longitudinal’ ethnography that aims to avoid the initial bias of the ‘observer effect’.^48^ We addressed this by asking participants to pick a habitual, common activity. In practice, the observations were more fluid and, in fact, contained several activities, some of these being incidental, such as transport or refreshment breaks.

Another limitation was that participants were sampled from the long-running EPIC-Norfolk study, which perhaps produced a particularly healthy, health-conscious pool of participants. In principle, reflective participants are desirable for qualitative research, but the transferability of our findings to the wider population might be more usefully considered in terms of learning from our participants’ thoughts and practical insights, rather than as necessarily reflecting population patterns in a representative way. In other words, while our participants experienced barriers to active living, such as osteoarthritis or widowhood that should resonate with older people elsewhere, they may have identified solutions more proactively than less health-conscious peers, and may, therefore, be somewhat unusual in this regard. Nevertheless, the solutions they found should be valued as practical and meaningful examples of participant-driven facilitators that others could adopt. A further caution about generalisability also relates to the setting of EPIC-Norfolk, given that even the experience of living in urban Norwich might not translate to urban environments in more deprived settings, or larger or more industrialised cities.

Finally, the sample was relatively homogenous by default (the EPIC-Norfolk sample is over 99% white English),^49^ but half the participants were from lower occupational classes, which is notably different than the majority of qualitative physical activity studies which have recruited largely middle-class participants.^50^ While we succeeded in obtaining a diverse sample of participants according to gender, social class, living status and location, we aimed for a degree of homogeneity to allow for a certain level of shared experience and saturation in our analysis. We therefore focused on the so-called third age, and excluded those aged over 80 years, who are more likely to grapple with different problems such as frailty. Biological age, however, turned out to be a relative construct: there were experiences of ill-health in the youngest age group in this study, and experiences of fitness or continued working life in the oldest age group.

## Conclusions

Levels of physical activity decline in older age, and older adults tend to find it difficult to meet recommendations for physical activity.^3^ ^5^ In this qualitative study, we interviewed older adults as ‘experts by experience’ and participated in their sedentary and physical activities to identify challenges as well as strategies for active living. With this study, we add empirical data and theoretical interpretations to recent reconceptualisations of health behaviours as social practices^35^ as a novel starting point for promoting healthy living. We suggest a need to pay more attention to the complex social context of active living and healthy ageing, rather than attending merely to individuals and their discrete behaviours. While we had a particularly motivated sample of participants whose views might have been shaped by long-time participation in a health-related study, their experiences can, nevertheless, serve as case studies that could be transformed into meaningful, ‘real-life’ interventions to reduce sedentary living, and integrate small bouts of purposeful physical activity into everyday life and its challenges.
